# Fairness requires deliberation: the primacy of economic over social considerations

**DOI:** 10.3389/fpsyg.2015.00747

**Published:** 2015-06-08

**Authors:** Guy Hochman, Shahar Ayal, Dan Ariely

**Affiliations:** ^1^Center for Advanced Hindsight, Social Science Research Institute, Duke University, Durham, NC, USA; ^2^Baruch Ivcher School of Psychology, Interdisciplinary Center Herzliya, Herzliya, Israel

**Keywords:** ultimatum game, cooperation, fairness, financial decision-making, dual-process, social utility, inequality aversion

## Abstract

While both economic and social considerations of fairness and equity play an important role in financial decision-making, it is not clear which of these two motives is more primal and immediate and which one is secondary and slow. Here we used variants of the ultimatum game to examine this question. Experiment 1 shows that acceptance rate of *unfair offers* increases when participants are asked to base their choice on their gut-feelings, as compared to when they thoroughly consider the available information. In line with these results, Experiments 2 and 3 provide process evidence that individuals prefer to first examine economic information about their own utility rather than social information about equity and fairness, even at the price of foregoing such social information. Our results suggest that people are more economically rational at the core, but social considerations (e.g., inequality aversion) require deliberation, which under certain conditions override their self-interested impulses.

## Introduction

The classical economic model suggests that individuals are rational, free of any extraneous considerations, and follow the principle of utility maximizing. Financial decisions are assumed to be entirely selfish and self-interested and no room is given for social motivations (von Neumann and Morgenstern, 1944; Becker, 1962; Kahneman et al., 1986). By contrast, psychological research suggests that even under this type of decisions cooperation is very much alive, and that individuals are more social oriented than portrayed by the basic rational economic model (Fehr and Schmidt, 1999).

The consistent deviations from the *Homo economicus* rational model (Fehr and Schmidt, 1999; Fehr and Gächter, 2000), provide ample evidence that financial decisions are governed by more than mere economic selfish motives (e.g., Cameron, 1999; Bolton and Ockenfels, 2000; Gino et al., 2009). One robust demonstration of this mix of motives is found in the ultimatum game, a bilateral bargaining setting which allows economic and social influences to be examined (Tabibnia et al., 2008). In the classical ultimatum game (Güth et al., 1982), one individual (the proposer) makes a proposal on how to divide a sum of money (the pie) with a second individual. The responder has then the opportunity to either accept the proposed division, in which case both players earn the amount proposed, or reject it, in which case both earn nothing. While the classical economic model suggests that proposers should offer the smallest possible amount, and responders should accept it, the typical results suggest that proposers tend to offer a fair share of 40–50% of the pie, and that the majority of respondents reject unfair (below 30%) offers (e.g., Güth et al., 1982; for a review, see Camerer and Thaler, 1999).

Thus, as in many other behavioral phenomena, evidence from the ultimatum game clearly supports the assertion that social considerations of equity and fairness, and not just selfish economic factors play an important role in financial decision-making. However, it is not yet clear which of the two is more primal and immediate and which one is secondary and slower (see, e.g., Lucas and Wagner, 2005). Which considerations, economic or social, are more primary, when we make complex financial decisions?

There has been a recent upsurge in theories that characterize human cognition and specifically choice behavior as governed by the interaction between two different systems (e.g., Epstein, 1994; Sloman, 1996; Kahneman, 2003; Bechara and Damasio, 2005; Evans, 2007). System 1, which is assumed to be fast, automatic, associative and emotionally charged, and System 2, which is assumed to be slow, deliberative, and affect-free. This dual-system approach leads to two contrasting models to describe the primacy of motives governing financial decision-making. The first model, suggests that we are more social in nature, but deliberative considerations make us more focused on economic considerations (Fehr and Schmidt, 1999). According to this model, which is more consistent with the psychological view, emotional considerations are the social and selfless elements, while deliberative considerations are the more analytical and focused on utility maximizing (Pillutla and Murnighan, 1996). By contrast, the second model suggests that economic self-interest is primal (Dawkins, 1990), but these motives are overridden by acquired social preferences for equity and altruism (Moore and Loewenstein, 2004; Henrich et al., 2005). According to this model, which is more consistent with the classical economic view, people’s basic considerations are driven by economic self-interest, and secondary considerations are deliberative and pro-social. Interestingly, the literature in behavioral decision making provides empirical evidence to support both of these models.

In support of the psychological point of view, recent neuroimaging studies suggest that unfair proposals in the ultimatum game are associated with negative emotional responses (Sanfey et al., 2003; Tabibnia et al., 2008). Similarly, it has been shown that individuals are more likely to reject unfair offers under tight time constraints (Sutter et al., 2003; Grimm and Mengel, 2011; Neo et al., 2013). Rand et al. (2012, 2013) showed that people cooperate more and thus demonstrate stronger tendency toward social motives when they are forced to think fast and use more automatic processes. Since emotional responses are assumed to be primary (Bechara and Damasio, 2005), and time pressure is assumed to inhibit deliberation (Usher et al., 2011; Russo et al., 2013), these results can be interpreted as supporting the view that people’s primary motives are social, and that their secondary motives are economic (e.g., Rand et al., 2013). In line with these claims, it has also been shown that non-human primates (e.g., Brosnan and de Waal, 2003; Brosnan, 2006) and infants (Hill and Sally, 2004) are highly sensitive to fairness and equity.

At the same time, however, it has also been shown that reliance on affective considerations lead proposers in the ultimatum game to make less generous offers than reliance on more computational (deliberative) considerations (Stephen and Pham, 2008). Similarly, it has been argued that inequality aversion (reflected by rejections of unfair offers) is a deliberative act, which relies on self-control (Knoch and Fehr, 2007). Thus, these results support the priority of economic elements proposed by the classical economic view and suggest that our primary motives are economically rational, and our secondary motives are more social and emotional. Consistent with this view, findings from studies employing tasks that are more analogous to the classic ultimatum game have shown that non-human primates (e.g., Jensen et al., 2007) and young children (Harbaugh et al., 2004) are more self-interested than adults.

In light of these contradictory and inconsistent findings, it is neither trivial nor clear which of these models best captures the priority we give to different considerations in our reasoning and behavior. In the current paper, we conducted three ultimatum game experiments that enabled us to juxtapose these two models and examine if the considerations of fairness and inequality aversion that influence people’s financial decision-making are more primary or secondary.

## Experiment 1: Gut Feeling versus Thorough Consideration

Experiment 1 used a modified version of a repeated trials ultimatum game under time constraints. In each trial, participants were instructed to make their decisions either quickly and based on their primary gut-feelings or to take their time and engage in a deliberate decision process. By confining them to these time constraints, we were able to examine choices based on rapid and primary motives as compared to secondary and slow motives (Usher et al., 2011; Russo et al., 2013).

### Method

#### Participants and Task

Fifty-one undergraduates from Duke University (average age = 22.0 years; 25 females) participated in the experiment as responders for monetary payoff. Participants received $5 as show up fee and a bonus payment according to their decisions in the game. Participants were told that they would play multiple trials with another participant via the computer. Actually, there were no real proposers, and all offers were made by the computer (programmed with Visual Basic 6.0). The experiment had 50 trials. The total pie in each trial was randomly sampled from the $10–$30 range (in $1 intervals). The split of the pie was 50:50, 60:40, 70:30, 80:20, or 90:10 (in favor of the proposer). Each participant got 10 offers for each split presented in random order.

Participants were seated in front of the computer screen, and were told that in each trial the proposer would be given an amount of money (that would vary from trial to trial), and would offer to split this amount. Participants were required to accept or reject each offer. To facilitate their decisions, the following information was available: the split of the total pie between them and the other player (in percentages), and the absolute value (in USD) of what was offered to them. To motivate their choices, participants were told that at the end of the experiment the computer would randomly select one of the trials in which they accepted the offer, and that they would be paid based on their allocated amount from that trial.

Participants were randomly assigned to one of two between-subjects conditions. In the time-pressure condition (*n* = 18), participants were instructed to respond to their gut feelings, and make their choices as fast as possible. In the no-time-pressure condition (*n* = 33), participants were instructed to consider all of the available information and to take their time before making their choice. To ensure compliance with these instructions, in the time-pressure condition participants were further informed that the computer would monitor their decision times, and that they would be prompted to answer faster if their responses were too slow (i.e., longer than 2 s). In the no-time-pressure condition, participants were further informed to take more time for deliberation if their responses were too fast (i.e., faster than 3 s). In addition, in both conditions participants were informed that 10% would be deducted from their final earnings each time they received a message that the time constraints were violated. The Ethics committee of Duke University approved this study.

### Results

The average response time was 0.73 s (SD = 0.19) in the time-pressure condition and 3.3 s (SD = 1.7) in the no-time-pressure condition. An independent-samples *t*-test revealed that this difference was significant [*t*(49) = 6.036, *p* < 0.0001]. These results validate our manipulation and suggest that the participants understood and adhered to the instructions to follow their gut-feelings or to examine all the available information (depending on the experimental condition).

**FIGURE 1:**
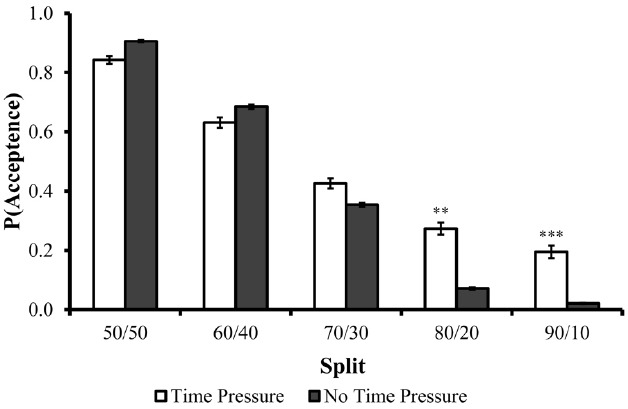
**Acceptance rates of each split as a function of the time constraint condition in Experiment 1.** Error bars are depicted in black lines. **p < 0.02, ***p < 0.0005.

The acceptance rates of each split as a function of the time constraint condition are illustrated in Figure 1. In line with previous findings, the majority of fair proposals (30% and above) in each phase were accepted, and acceptance rates decreased almost linearly as the offers became less fair. However, contrary to previous research (e.g., Sutter et al., 2003), when the offers were unfair, acceptance rates were much higher in the time-pressure condition than in the no-time-pressure condition (e.g., 27 versus 7% for an 80:20 split, and 19 versus 2% for a 90:10 split). No difference in acceptance rates was observed for fair proposals.

A 5 (offered split) × 2 (time constraint condition) repeated measures ANOVA revealed a significant effect for split [*F*(4,196) = 178.572, *p* < 0.0001] and a significant split × time-pressure interaction [*F*(4,196) = 6.495, *p* < 0.0001] on acceptance rates. No main effect was found for the time-pressure manipulation [*F*(1,49) = 1.43, *p* = 0.26]. Planned contrasts further revealed a significant difference between acceptance rate in the no-time-pressure and time-pressure conditions for the (unfair) 80/20 (*p* < 0.005) and 90/10 (*p* < 0.02) splits. These results suggest that encouraging primary considerations (gut-feelings) led to higher acceptance rates of unfair offers. Since social-interest considerations predict a decrease in acceptance rates when the offer is unfair (i.e., inequality aversion), the higher acceptance rates in the time-pressure condition compared to the no-time-pressure condition suggest that the economic motives are more primal and rapid whereas considerations of fairness and equity are more secondary and slow.

## Experiment 2: Pattern of Information Search

Experiment 1 showed that inequality aversion reflected by rejecting unfair offers in the ultimatum game reduced significantly when individuals were forced to respond rapidly. In Experiment 2 we aimed to further explore the underlying processes behind this behavior. The experiment used the same ultimatum game task as in Experiment 1, with a few modifications: participants were provided with all the possible information in the ultimatum game (i.e., the pie, the split, the allocated sum of the proposer, and the allocated sum to the responder), but only one piece of information was available at a time, and responders were required to select which information they wanted to see at each point in time, and in what order. Based on the mouselab method (Johnson et al., 1989), examining the order of information search enables to explore the information acquisition processes of decision makers. Assuming that people give priority to, and focus more on, the most important information (Payne et al., 1993), we used the order of information acquisition to assess the relative importance participants assigned to economic (personal gain) versus inequity (the split) information.

In addition, since the standard ultimatum game is based on a one-shot decision with no time constraints, we wanted to test the repeated-trials paradigm used in Experiment 1 without time constraints and see if it replicates traditional ultimatum game findings.

### Method

#### Participants and Task

Thirty-one undergraduates from Duke University (average age = 21.4 years; 19 females) participated in the experiment for monetary payoff. Participants received $5 as show up fee and a bonus payment according to their decisions in the game. The task was identical to the ultimatum game in Experiment 1, with the exception that the available information was the pie (i.e., the total available amount of money), the split of this amount between the participant and the other player, the absolute amount of money that would be allocated to the other player, and the absolute amount of money that was offered to the participant. However, all types of information were hidden, and were only displayed when participants pressed a button corresponding to the desired information. Participants were able to explore the available information as much as they wanted, and in any order, but they could only look at one piece of information at a time. The Ethics committee of Duke University approved this study.

To examine whether the repeated trials had an effect on acceptance rate, we included 60 trials that were divided into three blocks each consisting of 1/3 of the trials.

### Results

As in Experiment 1 the majority of fair proposals were accepted, and acceptance rates decreased as the offers became less fair. A 5 (offered split) × 3 (blocks of trials) repeated measures ANOVA revealed a significant main effect for split on acceptance rates [*F*(4,88) = 38.089, *p* < 0.0001], but not for blocks of trials [*F*(2,44) = 0.754, *p* = 0.48].^1^ Planned contrasts further demonstrated that there was no difference in acceptance rate between the first third of trials and the last third of trials (*p* = 0.66), or between the second third of trials and the last third of trials (*p* = 0.45). This pattern of results validates our design, as it concurs with previous findings that acceptance rate is highly dependent on fairness and not on material opportunism, and found no effect for repeated trials.

**FIGURE 2:**
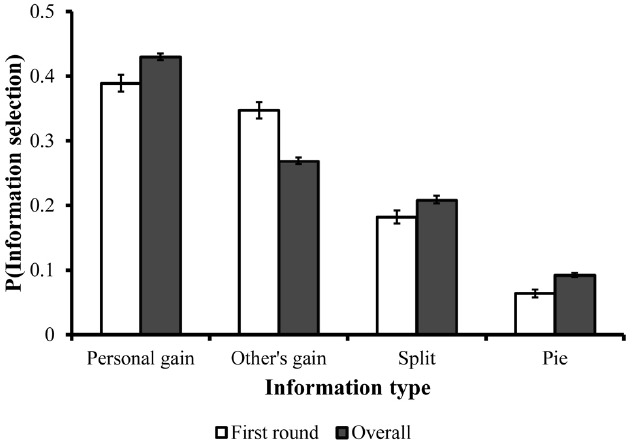
**Overall information search pattern and search pattern in the first phase in Experiment 2.** Error bars are depicted in black lines.

Next, to examine the order of information search, we analyzed the proportion of times in which each type of information was selected *first*, as well as the overall proportion for selecting each type of information. These results are illustrated in Figure 2. Across all trials participants preferred to examine personal-utility information first; namely information about what was allocated to themselves was selected first in 40% (SD = 40) of the times. By contrast, social-utility information about the split was selected first only in 18% (SD = 32) of the times. Information about what was allocated to the other person and the pie were selected first in 36% (SD = 39) and 6% (SD = 19) of the times, respectively. A repeated measures ANOVA revealed that these differences were significant [*F*(3,90) = 4.707, *p* < 0.005]. Similar results were obtained for the proportion of selecting each type of information across all information-search steps. Information about personal gains was selected in 43% (SD = 16) of the time, information about the gain of the other player 27% (SD = 15) of the time, information about the split 21% (SD = 18) of the time, and information about the pie 9% (SD = 11) of the time [*F*(3,90) = 20.036, *p* < 0.0001].

Since participants showed a high preference to examine information about their own gain first and only then social information about the split, these results support the assertion that the economic elements get priority over those of fairness and are considered as preliminary in the decision process. However, even though participants preferred to evaluate personal-utility information first, they later examined other types of information. Thus, it is unclear whether information-search patterns reflect solely the relative importance that individuals assign to personal-utility and social-utility information. For example, the tendency to first evaluate personal-utility information may reflect a mere preference for using information that is more related to self-interest to deduce other types of information (e.g., calculating the pie or the split based on information about their own and the other’s gains). Experiment 3 was designed to directly address this possibility as well as to further explore the underlying processes of the primary motives of economic decisions.

## Experiment 3: Limited Information

This experiment used the same ultimatum game task as in Experiment 2, with the exception that the only information available to participants was the absolute amount that was offered to them and the percentage split. While in one condition participants were required to choose the order of information acquisition, in the second condition they were required to only choose one of these two pieces of information. By confining the available information to either how much they will gain or how fair the offer is, we were able to directly examine whether participants preferred to base their decision solely on more economic or more social information.

### Method

#### Participants and Task

Forty-four undergraduates from Duke University (average age = 21.8 years; 25 females) participated in the experiment as responders for monetary payoff. Participants received $5 as show up fee and a bonus payment according to their decisions in the game. The task was identical to the ultimatum game in Experiment 2, with the exception that the only available information was the percentage split of the total amount between them and the other player and the absolute amount of money that was offered to them. In addition, participants were required to make their decisions as quickly as possible, and the time they spent on each type of information was recorded by the computer program.

Participants were randomly assigned to one of the two between-subjects conditions. In the sequential condition (*n* = 26), participants were required to select which information they wanted to see first and which to see second, but they could examine both types of information. In the choose-one condition (*n* = 18), participants were required to select which information they wanted to see, but they could only examine one type of information. The Ethics committee of Duke University approved this study.

### Results

**FIGURE 3:**
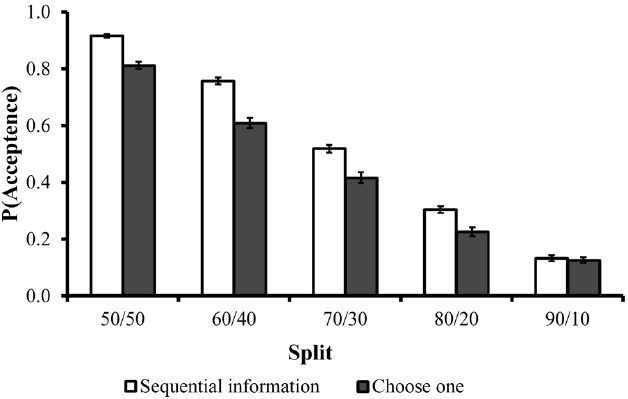
**Acceptance rates of each split as a function of experimental condition in Experiment 3.** Error bars are depicted in black lines.

The acceptance rates of each split are illustrated in Figure 3. As in Experiments 1 and 2, the majority of fair proposals were accepted, and acceptance rates decreased as the offers became less fair. A 5 (split) × 2 (sequential versus choose-one) repeated measures ANOVA revealed a significant main effect for split on acceptance rates [*F*(4,168) = 115.047, *p* < 0.0001], but not for the experimental condition [*F*(1,42) = 1.43, *p* = 0.24].

**FIGURE 4:**
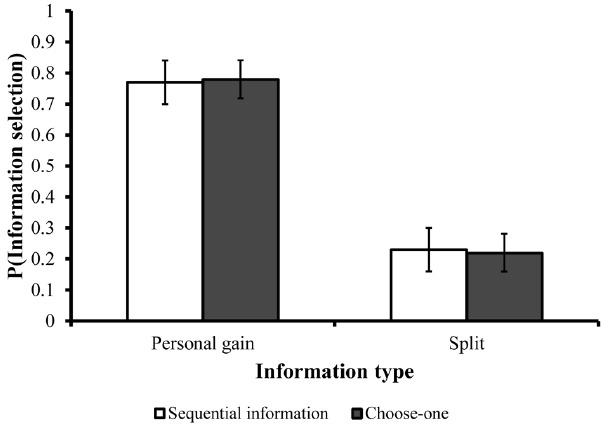
**Overall information search pattern and search pattern in the first phase in Experiment 3.** Error bars are depicted in black lines.

In the sequential condition, where participants were required to select which type of information they wanted to see first and which one second, personal-utility information (about what was offered to them) was selected first 77% (SD = 36) of the time. Similarly, in the choose-one condition, where only one type of information could be examined, most participants selected to examine only what was offered to them (78%, SD = 26). These results are illustrated in Figure 4. As can be seen, these results concur with the results of Experiment 1 and 2, and demonstrate that whether people have freedom to acquire all information or just part of it, they clearly give priority to economic information over fairness. This pattern supports the assertion that participants’ initial considerations are more selfish in nature whereas considerations for fairness and inequality aversion requires more time and deliberations.

In further support of this claim, we also found that in the choose-one condition, the average response time based on examining just the split (2.2 s, SD = 0.8) was almost twice as long as the response time based on examining the absolute value of the offer alone [1.2 s, SD = 0.4; *F*(1,9) = 6.868, *p* < 0.05]. Short response times are assumed to reflect reliance on instantly accessible information whereas long response times indicate more deliberate cognitive consideration (Ayal and Hochman, 2009). Thus, it appears that choices that were based on purely economic information were more rapid and primary, whereas choices that were based on information that is more social were more slow and secondary.

## General Discussion

Previous research suggests that both economic and social motives play an important role in financial decision-making (Cameron, 1999; Bolton and Ockenfels, 2000). In line with these observations, in this work we examined which of these motives is more primary and which is more secondary.

Experiment 1 showed that acceptance rates of unfair offers increased significantly when individuals based their decisions on their primary gut feelings. Since economic motives such as personal-utility lead to higher acceptance rates and social-utility motives such as fairness and inequality aversion lead to lower acceptance rates, these results provide evidence that economic motives are more rapid and primary, while social motives are more secondary and slow. In line with these results, Experiments 2 and 3 provide process evidence showing that individuals give priority to information about personal-utility rather than about fairness and inequality aversion, even at the price of foregoing this kind of social information. These findings might suggest that at the core, we are fundamentally self-interested and self-serving (Stephen and Pham, 2008), and that social considerations such as fairness are more deliberative and secondary (Knoch and Fehr, 2007). If this is true, it might be argued that on a broader perspective, the self-maximizing dogma of the classical economic view captures the primal component of the human essence and not necessarily our deliberate and calculated behavior.

The current data may also speak to the elusive role of neuropsychology and neuroeconomics in understanding the underlying psychological processes. Previous research suggests that fairness processing is accompanied by increased activation in brain regions associated with emotional and automatic reactions (Sanfey et al., 2003; Tabibnia et al., 2008), such as the anterior insula, the ventromedial prefrontal cortex (VMPFC), and the amygdala. One interpretation of these (and other related) findings is that the default (primary) response in financial decision-making is driven by social-utility considerations such as fairness (Sanfey et al., 2003; van’t Wout et al., 2005; Tabibnia et al., 2008). Our results seem at odds with this conclusion, as we show that the economic-selfish elements are primary, but that these considerations can be overridden by secondary social concerns (as reflected in participants’ final decisions). Interestingly, previous research suggests that the VMPFC may also reflect preferential engagement in personal-utility versus social-utility mentation (Ames et al., 2008; Mitchell, 2009). If this is the case, our results may point at an alternative interpretation. Specifically, the activity in the VMPFC might reflect the evaluation of primary personal-utility considerations. However, this evaluation might than be overridden by effective responses (via interaction with the amygdala and the anterior insula), which might stem from more deliberative and secondary social-utility considerations.

In terms of the dual-system models of reasoning, this approach is typically described by an automatic and primary processes that are biased and irrational and more delayed deliberative processes that are primarily logical and rational (e.g., Epstein, 1994; Evans, 2007). According to these accounts, deliberative processes are assumed to be activated to monitor and correct irrational automatic processes that are prone to errors (Kahneman, 2003; Masicampo and Baumeister, 2008). Here we show that this injective function which equates primary with irrational choice behavior and deliberative with rational behavior does not always stands. As our results demonstrate, there are certain situations in which following one’s gut-feeling or acting impulsively appear to be the rational “cold” behavior which is accompanied by subsequent social and more “hot” considerations (see also Ayal et al., 2011; under review; Usher et al., 2011). Interestingly, the fact that in their final choices, people were highly sensitive to fairness and inequality aversion suggests that contrary to what was previously assumed, the deliberative system monitors and correct primary considerations, even when these initial considerations are more in line with the traditional rational recommendation.

Finally, the current study offers some insights into the ways in which affective and cognitive inputs are integrated during the decision process. Typically, intuition is assumed to operate on affective information whereas deliberation is linked to more cognitive and rational considerations. In support of this view, Small et al. (2007) showed that judgment based on emotionally-charged inputs (e.g., an identifiable victim) is more altruistic and others-interested, whereas more deliberative judgment based on neutral, and unbiased inputs (e.g., statistical information about victims) is more “rational” and self-interested. Here we provide a qualification to these findings by showing that the output of the cognitive system is highly dependent on the inputs to the system and not only on the type of system that processes it (i.e., intuitive or deliberate). Specifically, it is possible that when people consider neutral, emotion-free, highly relevant and unbiased information (e.g., monetary payoffs from an unknown proposer in the ultimatum game), our gut-feelings are logical, cold, and rational. In contrast, when the input is emotionally-charged and involve information that cannot be easily quantified (e.g., experienced-emotions), the same initial gut reaction might be less rational and susceptible to wide variety of cognitive biases.

### Conflict of Interest Statement

The Editor Panagiotis Mitkidis declares that, despite being affiliated to the same institution as the authors Guy Hochman and Dan Ariely, the review process was handled objectively and no conflict of interest exists. The authors declare that the research was conducted in the absence of any commercial or financial relationships that could be construed as a potential conflict of interest.

## Footnotes

1 A virtually identical pattern of acceptance rates was observed when we only analyzed the first trial for each participant.
